# Oral Glucose Tolerance Test Performed after 28 Gestational Weeks and Risk for Future Diabetes—A 5-Year Cohort Study

**DOI:** 10.3390/jcm12186072

**Published:** 2023-09-20

**Authors:** Esther Maor-Sagie, Mordechai Hallak, Yoel Toledano, Rinat Gabbay-Benziv

**Affiliations:** 1 Department of Obstetrics and Gynecology, Hillel Yaffe Medical Center, Hadera 3820302, Israel; estimaorsagie@gmail.com (E.M.-S.); mottih@hymc.gov.il (M.H.); 2 Meuhedet HMO, Rehovot 7610001, Israel; yoel.t@meuhedet.co.il; 3 The Ruth and Bruce Rappaport Faculty of Medicine, Technion—Israel Institute of Technology, Haifa 3200003, Israel

**Keywords:** diabetes mellitus, gestational diabetes, prediction, pregnancy, oral glucose tolerance test

## Abstract

Gestational diabetes mellitus (GDM) is diagnosed by an oral glucose tolerance test (oGTT), preferably performed at 24 + 0–28 + 6 gestational weeks, and is considered a risk factor for type 2 diabetes (T2DM). In this study, we aimed to evaluate the risk of T2DM associated with abnormal oGTT performed after 28 weeks. We conducted a retrospective cohort study that included parturients with available glucose levels during pregnancy and up to 5 years of follow-up after pregnancy. Data were extracted from the computerized laboratory system of Meuhedet HMO and cross-tabulated with the Israeli National Registry of Diabetes (INRD). The women were stratified into two groups: late oGTT (performed after 28 + 6 weeks) and on-time oGTT (performed at 24 + 0–28 + 6 weeks). The incidence of T2DM was evaluated and compared using univariate analysis followed by survival analysis adjusted to confounders. Overall, 78,326 parturients entered the analysis. Of them, 6195 (7.9%) performed on-time oGTT and 5288 (6.8%) performed late oGTT. The rest—66,846 (85.3%)—had normal glucose tolerance. Women who performed late oGTT had lower rates of GDM and T2DM. However, once GDM was diagnosed, regardless of oGTT timing, the risk of T2DM was increased (2.93 (1.69–5.1) vs. 3.64 (2.44–5.44), aHR (95% CI), late vs. on-time oGTT, *p* < 0.001 for both). Unlike in oGTT performed on time, one single abnormal value in late oGTT was not associated with an increased risk for T2DM.

## 1. Introduction

Gestational diabetes (GDM) is one of the most common complications during pregnancy, with short- and long-term implications for the mother, fetus, and offspring [1]. However, despite large-scale studies, major controversies remain about its diagnosis, treatment, and future implications.

The American College of Obstetrics and Gynecology (ACOG) recommends screening all pregnant women for GDM, preferably at 24 + 0 to 28 + 6 gestational weeks [1,2]. In the United States, the preferred method for screening is the two-steps approach, which uses the glucose challenging test (GCT) followed by a diagnostic 3 h 100 g oral glucose tolerance test (oGTT) for the screen-positive women [1]. The timing of oGTT between 24 and 28 weeks of pregnancy was chosen to align with the physiological changes of pregnancy, maximizing the number of chances to detect GDM and allow for timely intervention and management.

Notably, over the last years, several publications investigated the importance of late GDM, diagnosed in the third trimester after 28 gestational weeks. In a Dutch cohort study, the authors reported GDM diagnoses in 23.5% of parturients who initially tested negative at 24–28 gestational weeks [3]. Similarly, other studies demonstrated around 25% late GDM diagnoses in women who underwent late oGTT because of suspected large-for-gestational-age fetuses or polyhydramnios [4,5,6], with even higher rates of GDM for women with obesity [5,6].

Several studies evaluated the clinical implications of late GDM [5,7,8,9,10], emphasizing short-term maternal and neonatal outcomes with conflicting results, especially regarding the delivery of large-for-gestational-age babies and mode of delivery. To note, there was great variance among studies regarding the population, the methodology, the definition of late GDM, the GDM screening approach, and the evaluated outcomes; therefore, conclusions were hard to draw.

Regardless of that, GDM is a well-established risk factor for type 2 diabetes mellitus (T2DM). It is estimated that up to 70% of women with GDM will develop T2DM 22–28 years after pregnancy [1]. To the best of our knowledge, none of the studies that investigated late oGTT evaluated the risk of future T2DM based on the timing of GDM diagnosis during pregnancy. Thus, in this study, we aimed to investigate the risk of T2DM in women who performed abnormal late oGTT during pregnancy in a large cohort of women with 5 years of follow-up after pregnancy.

## 2. Materials and Methods

A retrospective cohort study aimed to evaluate the prediction performance of late 100 g oGTT during pregnancy for T2DM in a 5-year follow-up. The study included all women with documented singleton pregnancies (by pregnancy registry) without diabetes diagnosis, with last menstrual period (LMP) between 1 January 2017 and 31 December 2020. Pregnancies complicated by early GDM, defined as fasting plasma glucose at early pregnancy at or above 92 mg/dL, or women who performed oGTT at less than 24 gestational weeks were excluded. For women with more than one pregnancy during the study period, only the first pregnancy was included to ensure the longest available follow-up time. On-time oGTT was defined as oGTT performed between 24 + 0 and 28 + 6 weeks. Late oGTT was defined as oGTT performed after 28 gestational weeks. Risk for T2DM was compared between women with abnormal oGTT results—either single abnormal value (SAV) or GDM and, according to oGTT timing, on-time oGTT vs. late oGTT. Follow-up time was defined as the date of diabetes diagnosis, the date of data extraction (13 November 2022), or death—whichever came first. The study was approved by the local Institutional Review Board committee (10-18-08-21). Due to the retrospective nature of the study, informed consent was waived.

For this study, data were extracted from a dataset encompassing more than 5 years of laboratory data collected by Meuhedet HMO (health maintenance organization), cross-tabulated with a pregnancy registry, and integrated with the Israeli National Diabetes Registry (INDR). Meuhedet is one of the four Israel health insurance and medical services organizations to which Israeli residents must belong under Israel’s universal healthcare framework. Maternal data included maternal age, body mass index (BMI), and diagnosis of hypertension. Delivery data included gestational age at delivery and neonatal gender. All clinical data were retrieved from the parturient electronic medical records at the time of pregnancy. Laboratory data included first-trimester fasting glucose levels, 50 g glucose challenge test (GCT), and 100 g oGTT values. T2DM diagnosis was retrieved from the INDR. As previously described [11], since 2012, all health medical organizations in Israel are required by law to report cases of diabetes to the INDR. Data in this registry were linked to the pregnancy registry and the laboratory data of Meuhedet. Diabetes diagnosis is updated daily to the registry and defined as meeting one or more of the following criteria: (1) glycated hemoglobin greater than or equal to 6.5% (47.5 mmol/mol), (2) serum glucose concentrations greater than or equal to 200 mg/dL (11.1 mmol/L) in 2 tests performed at an interval of at least 1 month, and (3) 3 or more purchases of glucose-lowering medications. The registry has a sensitivity of 95% and the positive predictive value is 93%.

By convention, and according to Israeli guidelines, all parturients are recommended to undergo fasting plasma glucose level in the first trimester to exclude overt diabetes (>125 mg/dL). Screening for GDM is recommended for all women at 24–28 gestational weeks by the two-steps approach. Late oGTT, after 28 weeks, is usually performed for latecomers or as part of large-for-gestational-age or polyhydramnios evaluation. Threshold values for GDM are consistent throughout pregnancy and defined according to the Carpenter and Coustan values [2], which require a GDM diagnosis to include at least two out of four abnormal values.

### Statistical Analysis

At first, we utilized univariate analysis to evaluate differences between women who performed on-time oGTT, late oGTT, or had normal glucose tolerance. Also, we evaluated differences according to oGTT results: between women with SAV or GDM at on-time oGTT and women with SAV or GDM at late oGTT. We determined all women in the cohort without SAV or GDM diagnosis (including women with normal GCT or women with four normal values on oGTT) as women with normal glucose tolerance (control group). Maternal age and BMI were evaluated both as continuous variables and as categorical variables (with a cutoff of 35 and 40 years for age and 30 kg/m^2^ for BMI). Glucose levels, gestational age at delivery, and time to follow-up were treated as continuous variables, while hypertension, GDM, neonatal gender, and T2DM were treated as categorical variables. Categorical variables were compared using χ^2^ tests, and the Kruskal–Wallis test was used to test differences for continuous variables. All the tests were 2-tailed and *p* < 0.05 was considered statistically significant. Next, to account for different follow-up times, we computed Kaplan–Meier hazard curves, applied Cox regression analysis, and determined the Hazard ratio (HR) with a 95% confidence interval (CI) for the cumulative incidence of T2DM, with maternal age, BMI, and maternal hypertension as covariates, using the control group as the reference group.

Lastly, due to the large impact of obesity on future T2DM, the risk for T2DM according to oGTT results was stratified according to maternal pre-pregnancy BMI and divided into women with obesity (BMI ≥ 30 kg/m^2^) and without obesity (BMI < 30 kg/m^2^).

## 3. Results

### 3.1. Study Population

**Figure 1:**
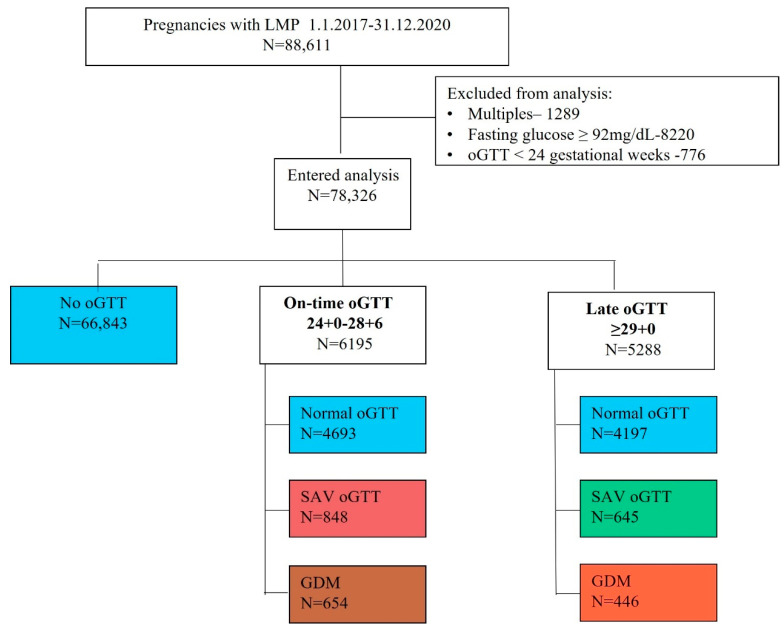
Study cohort. LMP—last menstrual period; oGTT—oral glucose tolerance test; GDM—gestational diabetes; SAV—single abnormal value.

Our dataset included 88,611 women with LMP during the study period and T2DM data. After excluding all multiple pregnancies (n = 1289), women with first-trimester fasting plasma glucose levels ≥ 92 mg/dL (n = 8220), and women with oGTT performed prior to 24 weeks gestation (n = 776), we were left with 78,326 women who were eligible for analysis. Of them, 6195 (7.9%) women performed oGTT at 24 + 0–28 + 6 gestational weeks (on-time oGTT) and 5288 (6.8%) performed oGTT after 28 gestational weeks (late oGTT). Demographics, baseline characteristics, glucose values, and rates of T2DM according to the timing of oGTT are presented in Table 1 and Figure 1. Overall, women who performed late oGTT had lower rates of abnormal oGTT results and lower rates of T2DM during the study period. Maternal variables and risk for T2DM are further presented, stratified by oGTT results when performed on time and late in gestation (Table 2). Regardless of oGTT timing, women with GDM were older and had higher obesity levels compared to women with normal glucose tolerance. Moreover, their glucose values throughout pregnancy were higher compared to women with SAV oGTT or women with normal glucose tolerance (Table 2).

### 3.2. Incidence of Type 2 Diabetes

In the univariate analysis, for women who performed late oGTT, GDM diagnosis was associated with higher rates of T2DM compared to SAV oGTT or normal glucose tolerance. Nevertheless, unlike women with on-time oGTT, SAV results on late oGTT were not associated with an increased risk for T2DM compared to women with normal glucose tolerance.

**Figure 2:**
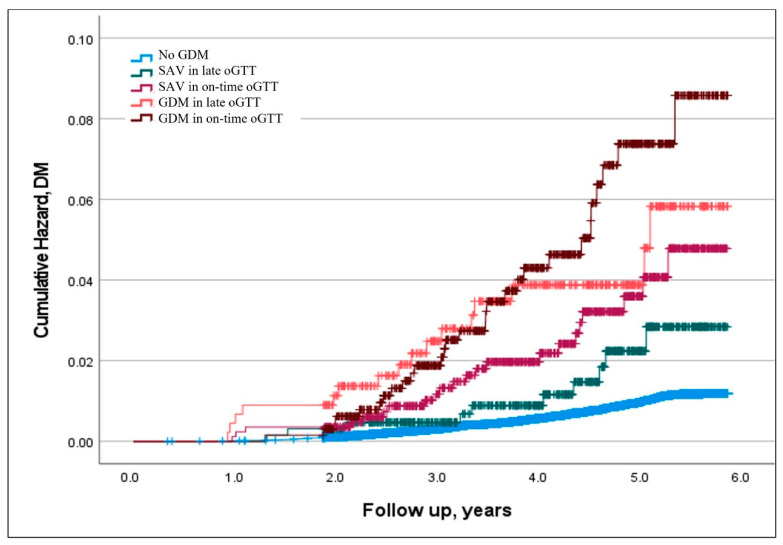
Abnormal oGTT and cumulative incidence of diabetes. Legend: Censored Kaplan—Meier Hazard curves for type 2 diabetes mellitus plotted for normal glucose status, single abnormal value oGTT, and GDM diagnosed at on-time oGTT (performed at 24 + 0–28 + 6 weeks) and late oGTT (performed after 28 weeks). oGTT—100 g oral glucose tolerance test; GDM—gestational diabetes; DM—diabetes mellitus; SAV—single abnormal value.

Using the Cox-regression survival analysis, and adjusted to maternal age, BMI, and hypertension, abnormal oGTT results, either SAV or GDM, that were diagnosed from on-time oGTT (24–28 gestational weeks) indicated a higher risk for T2DM compared to SAV or GDM that was diagnosed after 28 gestational weeks (Table 3, Figure 2).

### 3.3. Stratification by Obesity Status

**Figure 3:**
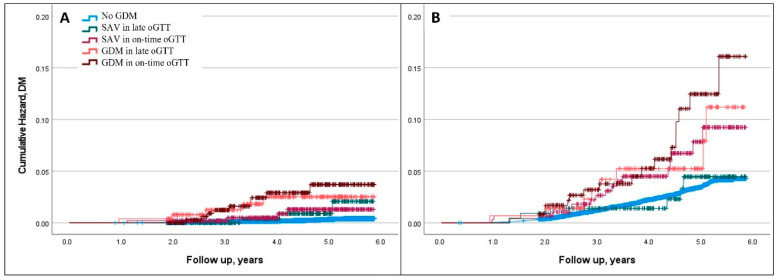
Abnormal oGTT and cumulative incidence of diabetes for women with and without obesity. Legend: Censored Kaplan–Meier Hazard curves for type 2 diabetes mellitus plotted for normal glucose status, SAV oGTT, and GDM diagnosed at on-time oGTT (performed at 24 + 0–28 + 6 weeks) and late oGTT (performed after 28 weeks). Curves are constructed and presented separately for women with and without obesity. (**A**) BMI < 30 kg/m^2^; (**B**) BMI ≥ 30 kg/m^2^; oGTT—100 g oral glucose tolerance test; GDM—gestational diabetes; DM—diabetes mellitus; SAV—single abnormal value.

Due to the significant association between BMI and T2DM, which was also evident in our cohort, we repeated the analysis separately for women with and without obesity (Table 4 and Table 5, Figure 3). The absolute incidence of T2DM was higher for women with obesity. That being said, their aHR that was solely related to abnormal oGTT results was lower compared to women without obesity.

## 4. Discussion

In this study, we aimed to investigate the risk of T2DM, over 5 years of follow-up, among women with abnormal oGTT results performed after 28 weeks of gestation (late oGTT) as compared to women who had abnormal oGTT results at 24–28 weeks of gestation (on time oGTT) and to women with normal glucose tolerance.

Our study results demonstrate the following findings: a. Women who perform late oGTT have lower rates of GDM and T2DM; b. Once GDM is diagnosed, regardless of oGTT timing, the risk of T2DM is increased even in a 5-year follow-up; c. The risk of T2DM following GDM diagnosis at late oGTT is increased for women with and without obesity; d. A SAV oGTT is associated with T2DM only if oGTT was performed prior to 28 gestational weeks.

### 4.1. Results in the Context of What Is Known

GDM is considered a well-established risk factor for the development of T2DM [1,2]. However, since the majority of GDM cases are diagnosed between 24 + 0 and 28 + 6 weeks, none of the studies evaluated the risk of T2DM specifically when GDM was diagnosed later in gestation.

Prior studies that evaluated the clinical implication of late oGTT investigated the maternal and neonatal short-term outcomes with conflicting results [5,7,10]. Thus, there is no consensus on yield and indications for performing oGTT in late pregnancy. Our results demonstrated that women who performed late oGTT had a lower prevalence of abnormal oGTT results, both SAV and GDM. This result is probably related to the different maternal characteristics and the indications for performing oGTT. Women who performed late oGTT were younger, with lower rates of hypertension and lower glucose levels in the first trimester and at GCT compared to women who performed on-time oGTT. Moreover, although indications for late oGTT were unavailable for this dataset, we assume that indications included latecomers or women with large-for-gestational-age fetuses or polyhydramnios with a previous normal GCT screening. Accordingly, this group represents women with lower pre-pregnancy metabolic risk and, therefore, lower abnormal oGTT risk and lower risk for T2DM.

According to our results, once GDM was diagnosed, regardless of the timing of the oGTT, the risk for T2DM was about three folds higher compared to women with normal glucose tolerance during gestation. Nevertheless, women with only a SAV oGTT had a statistically significant increased risk for T2DM only if oGTT was performed between 24 + 0 and 28 + 6 gestational weeks. Several possibilities can explain this difference. First, insulin resistance during pregnancy is mainly increased from 16 to 26 weeks of gestation, with a mild increase thereafter [12]. It is possible that late GDM, diagnosed to a large extent after normal GCT screening, represents milder insulin insensitivity when compared to on-time oGTT; therefore, when using the same thresholds for diagnosis, abnormal oGTT results will be associated with lower rates of T2DM compared to on-time oGTT. This is supported by the fact that SAV in late oGTT, unlike in on-time oGTT, was not statistically associated with an increased risk of T2DM in our cohort. These women may be prone to T2DM later in life, and our limited 5-year follow-up was too short to detect the risk. A second possible explanation considers the need for different thresholds for GDM diagnosis at a more advanced stage of pregnancy. O’Sullivan, who set the first thresholds for GDM, tested women in their second and third trimesters [13]. Further studies have tried to determine the correct thresholds during the third trimester regarding fetal outcomes [14]. The authors compared the glucose levels by home glucose monitoring between 2 groups of women who conducted oGTT after 33 weeks due to risk factors. One group was diagnosed with GDM and the other was negative. They found that the distribution of glucose values between the groups was significantly different nevertheless, with overlapping. Women who were diagnosed and treated reached lower glucose values in the third trimester and had lower rates of macrosomia and cesarean deliveries. These results were explained by the continuity effect of hyperglycemia. The authors concluded that high-risk women who do not fulfill the criteria for GDM diagnosis should be treated with the same attention as GDM parturients due to their risk factors and that no different thresholds should be set. Lastly, it is possible that the reproducibility of the oGTT decreases with advanced gestational age; therefore, there are more false positive and negative results for late oGTT [15].

### 4.2. Obesity and Type 2 Diabetes Incident

The underlying mechanism for T2DM is thought to be different among women with and without obesity [16]. A common underlying mechanism for GDM development is relative pancreatic insufficiency or β-cell dysfunction, which is possibly the predominant mechanism in women with normal BMI. Advanced gestation insulin resistance increases in order to preserve fetal demands, leading to accelerating ongoing pancreatic β-cell exhaustion and an increased risk of postpartum T2DM. Alternatively, in women with obesity, excessive adiposity may promote a proinflammatory state and insulin resistance, which contribute to both GDM development and later T2DM [17]. 

Prior studies that evaluated combinations of risk factors and postpartum dysglycemia [16] demonstrated that GDM alone had a comparable risk for T2DM, such as having two risk factors—obesity or post-delivery weight retention, for example. Having GDM on top of these risk factors exacerbates the effect of GDM on T2DM development. Concordant with that observation, our results showed that women with obesity had a significantly higher risk of developing T2DM compared to women without obesity. That being said, GDM diagnosis increased the risk of T2DM in women with and without obesity.

### 4.3. Research Implications

Our results emphasize the need to set universal standards for late oGTT, regarding indications, thresholds of diagnosis, and treatment advantage. Further studies should focus on both short-term and long-term outcomes for the mother and offspring and the extent of improvement in outcomes.

Since hyperglycemia is a continuum throughout pregnancy, and several studies have demonstrated increased fetal and maternal risk with increasing glucose levels even within the normal range [18,19], we need models with a longer follow-up time to determine the actual risk of late GDM and SAV diagnosis. Understanding the long-term implications of the late GDM diagnosis might clarify the underlying mechanism and contribute to the follow-up and therapeutic approach to these patients.

### 4.4. Clinical Implications

Our results imply that GDM diagnosed in late gestation represents a true metabolic disturbance and is not just a matter of threshold. This may imply its significance when managing GDM diagnosed in late gestation. Moreover, our results suggest that women with late GDM diagnoses should be further evaluated for risk factors and be tested for diabetes postpartum according to the ADA guidelines [2] in a similar way to women diagnosed with GDM at 24 + 0 and 28 + 6 gestational weeks. Regarding SAV, our findings do not support the need for GDM follow-up postpartum for women with late SAV solely based on this diagnosis. However, as long as we continue to diagnose late GDM with the same thresholds, we should treat these women with caution and recruit them to follow up according to their risk factors, regardless of their GDM status.

### 4.5. Strength and Limitations

The strengths of this study include the large cohort and the linkage of two detailed databases with systematic data collection measured rather than reported. Our results were based directly on the laboratory glucose values and INDR solid criteria and not on reported GDM or T2DM diagnosis. To avoid prediabetes patients, we only included women with fasting glucose less than 92 mg/dL and women with oGTT performed after 20 weeks. Nevertheless, our study was not free of limitations, mainly due to its retrospective nature. First, the Meuhedet HMO pregnancy registry has been limited to the last five years. Second, we lacked data on other covariates, such as a family history of T2DM or possible after-pregnancy interventions such as weight reduction or lifestyle modifications that might have interfered with the risk for T2DM. Third, we did not have the indication for late oGTT performance or if an oGTT was performed for the second time during pregnancy. Lastly, the INRD does not include prediabetic state and T2DM diagnosis by 75GR oGTT; however, only a few participants went through this test, and we assume all of them were captured by the INRD diagnostic criteria postpartum.

## 5. Conclusions

When diagnosed after 28 weeks, GDM, but not SAV, is associated with an increased risk of T2DM over 5 years of follow-up. Further studies are needed to standardize late oGTT regarding indications, thresholds for diagnosis, short- and long-term implications, and yield for treatment.

## Figures and Tables

**Table 1:** Baseline demographic and future type 2 diabetes stratified by timing of oGTT.

	No oGTT N = 66,843	on-Time oGTT N = 6195	Late oGTT N = 5288	*p* Value
Maternal age, years	28.2 (24.0–33.2)	31.9 (27.4–36.1)	30.4 (25.9–35.4)	**<0.001**
Age ≥ 35 years	12,328 (18.4)	1943 (31.4)	1453 (27.5)	**<0.001**
Age ≥ 40 years	3372 (5.0)	568 (9.2)	405 (7.7)	**<0.001**
BMI kg/m^2^	24.2 (21.5–27.8)	26.3 (23.1–30.6)	26.3 (23.1–30.6)	**<0.001**
BMI ≥ 30	9181 (16.8)	1667 (29.8)	1407 (29.7)	**<0.001**
Hypertension	478 (0.7)	101 (1.6)	54 (1)	**<0.001**
First-trimester fasting glucose, mg/dL	8 (76–85)	82 (78–86)	81 (76–85)	**<0.001**
GCT, mg/dL	97 (82–117)	151 (143–162)	133 (102–150)	**<0.001**
Fasting oGTT, mg/dL	--	77 (72–83)	76 (71–82)	**<0.001**
1 h oGTT, mg/dL	--	144 (119–170)	144 (122–166)	**0.553**
2 h oGTT, mg/dL	--	115 (96–138)	116 (98–137)	**0.605**
3 h oGTT, mg/dL	--	85 (65–104)	87 (67–105)	**0.007**
oGTT week	--	26.6 (25.6–27.7)	32.7 (30.4–35.6)	**<0.001**
oGTT results				
SAV oGTT	--	848 (13.7)	645 (12.2)	
GDM	--	654 (10.6)	446 (8.4)	**<0.001**
Gestational age at delivery, weeks	39.7 (38.3–40.7)	39.6 (38.6–40.6)	39.9 (38.9–40.7)	**<0.001**
Baby sex, male ^@^	26,545 (50.7)	3147 (53.7)	2736 (54.5)	**<0.001**
Follow-up time	4.4 (3.4–5.2)	4.3 (3.3–5.0)	4.4 (3.4–5.1)	**<0.001**
Type 2 DM, cumulative				
1- Year T2DM	10 (0)	1 (0)	3 (0.1)	**0.091**
2- Year T2DM	77 (0.1)	12 (0.2)	9 (0.2)	**0.156**
3- Year T2DM	186 (0.3)	42 (0.7)	23 (0.4)	**<0.001**
4- Year T2DM	304 (0.5)	75(1.2)	36 (0.7)	**<0.001**
5- Year T2DM	456 (0.7)	110(1.8)	53 (1)	**<0.001**

Data are presented as median (IQR) for continuous variables and n (%) for categorical values. Significant differences are presented in bold (*p* < 0.05). SAV—defined as one abnormal value on oGTT (Carpenter and Coustan threshold values)^2^; GDM—defined as two abnormal values on oGTT (Carpenter and Coustan thresholds values)^2^; ^@^ gender results available for 63,261 deliveries. BMI—body mass index; oGTT—100 g oral glucose tolerance test; SAV—single abnormal value; GDM—gestational diabetes; T2DM—type 2 diabetes mellitus; GCT—glucose challenge test.

**Table 2:** Baseline demographic and future type 2 diabetes stratified by oGTT results.

	Normal Glucose Tolerance N = 75,733	SAV Late oGTT N = 645	SAV On-Time oGTT N = 848	GDM Late oGTT N = 446	GDM On-Time oGTT N = 654	*p* Value
Maternal age, years	28.6 **^a,b,c,d^** (24.1–33.5)	31.8 **^e,g^** (27.1–36.1)	32.6 (28.4–37.1)	31.7 (27–37.3)	33.2 (28.7–37.1)	**<0.001**
Age ≥ 35 years	14,772 **^a,b,c,d^** (19.5)	211 **^g^** (32.7)	311 (36.7)	169 (37.9)	261 (39.9)	**<0.001**
Age ≥ 40 years	4061 (5.4) **^a,b,c,d^**	57 (8.8) **^f^**	96 (11.3)	60 (13.5)	71 (10.9)	**<0.001**
BMI kg/m^2^	24.4 **^a,b,c,d^** (21.7–28.1)	27.4 (23.4–32.2)	27.4 (23.8–32)	27.1 (23.8–31.4)	27.7 (32–24)	**<0.001**
BMI ≥ 30	11,363 **^a,b,c,d^** (18.1)	21 8(38)	292 (37.5)	143 (35.3)	239 (38.8)	**<0.001**
Hypertension	588 (0.8) **^b,d^**	3(0.5) **^e,g^**	17(2)	7(1.6)	18(2.8)	**<0.001**
First-trimester fasting glucose, mg/dL	80 (46–80) **^a,b,c,d^**	82 (78–86) **^g^**	83 (79–87) **^i^**	82 (78–86) **^j^**	84 (80–88)	**<0.001**
GCT, mg/dL	99 **^a,b,c,d^** (83–117)	145 **^e,f,g^** (129–157)	155 **^h,i^** (146–166)	151 **^j^** (136–169)	162 (150–175)	**<0.001**
Fasting oGTT, mg/dL	76 **^a,b,c,d^** (71–81)	80 **^e,f,g^** (74–86.5)	81 **^h,i^** (75–87)	83 (76–92)	83 (76–93)	**<0.001**
1 h oGTT, mg/dL	135 **^a,b,c,d^** (114–154)	181 **^f,g^** (161–190)	180 **^h,i^** (165–191)	195 (185–210)	195 (185–211)	**<0.001**
2 h oGTT, mg/dL	108 **^a,b,c,d^** (93–125)	144 **^f,g^** (125–159)	143 **^h,i^** (122.3–157)	171 (159.8–188)	171 (160–186)	**<0.001**
3 h oGTT, mg/dL	82 **^a,b,c,d^** (64–99)	98 **^e,f,g^** (72–118.5)	98 **^h,i^** (72–116)	113 (88.8–140)	117 (86.8–143)	**<0.001**
oGTT week	28.6 **^a,b,c,d^** (26.4–32.6)	32.1 **^e,g^** (30.3–34.7)	26.7 **^h^** (25.7–27.7)	32.1 **^j^** (30.1–34.5)	26.7 (25.6–27.7)	**<0.001**
Gestational age at delivery, weeks	39.7 **^a,b,c,d^** (38.4–40.7)	39.7 (38.7–40.4)	39.3 (38.4–40.3)	39.3 (38.3–40.1)	39 (38.1–39.9)	**<0.001**
Baby sex, male ^@^	31,147(51.2)	336(55.1)	420(52.4)	225(53.1)	300(49)	0.212
Follow-up time	4.4 (3.4–5.2) **^b,c,d^**	4.3 (3.2–5.1) **^f,g^**	4.2 (3.2–5)	4.1 (2.9–5)	4 (3–4.9)	**<0.001**
Type 2 DM, cumulative						
1-Year T2DM	11 (0) **^c^**	0 (0) **^f^**	1 (0.1) **^h^**	2 (0.4) **^j^**	0 (0)	**<0.001**
2-Year T2DM	84 (0.1) **^c,d^**	2 (0.3) **^f^**	3 (0.4) **^h^**	5 (1.1)	4 (0.6)	**<0.001**
3-Year T2DM	218 (0.3) **^b,c,d^**	3 (0.5) **^f,g^**	9 (1.1) **^h^**	10 (2.2)	11 (1.7)	**<0.001**
4-Year T2DM	361 (0.5) **^b,c,d^**	5(0.8) **^f,g^**	14 (1.7) **^h,i^**	14 (3.1)	21 (3.2)	**<0.001**
5-Year T2DM	542 (0.7) **^b,c,d^**	10(1.6) **^f,g^**	22 (2.6) **^i^**	16 (3.6)	29 (4.4)	**<0.001**

Data are presented as median (IQR) for continuous variables and n (%) for categorical values. Significant differences are presented for comparison between all groups; column “*p*-value”, significant in bold (*p* < 0.05) and for comparison between every 2 groups: a—between normal glucose tolerance and SAV on late oGTT; b—between normal glucose tolerance and SAV on on-time oGTT; c—between normal glucose tolerance and GDM on late oGTT; d—between normal glucose tolerance and GDM on on-time oGTT; e—between SAV on late oGTT and SAV on on-time oGTT; f—between SAV on late oGTT and GDM on late oGTT; g—between SAV on late oGTT and GDM on on-time oGTT; h—between SAV on on-time oGTT and GDM on late oGTT; i—between SAV on on-time oGTT and GDM on on-time oGTT; j—between GDM on late oGTT and GDM on on-time oGTT. The “normal glucose tolerance” group includes women with normal GCT or oGTT. SAV—defined as one abnormal value on oGTT (Carpenter and Coustan threshold values)^2^; GDM—defined as two abnormal values on oGTT (Carpenter and Coustan thresholds values)^2^; ^@^ gender results available for 63,261 deliveries. BMI—body mass index; oGTT—100 g oral glucose tolerance test; SAV—single abnormal value; GDM—gestational diabetes; T2DM—type 2 diabetes mellitus; GCT—glucose challenge test.

**Table 3:** Cox-regression analysis for the development of type 2 diabetes mellitus according to the study groups.

	aHR	95% CI	*p* Value
Maternal age, years	1.046	1.031–1.061	<0.001
BMI ≥ 30	8.871	7.270–10.827	<0.001
Maternal hypertension	1.843	1.099–3.091	0.021
Normal glucose tolerance	***		
SAV late oGTT	1.323	0.682–2.563	0.408
SAV on-time oGTT	2.139	1.362–3.360	<0.001
GDM late oGTT	2.933	1.685–5.105	<0.001
GDM on-time oGTT	3.642	2.441–5.436	<0.001

*** oGTT at 24 + 0–28 + 6 gestational weeks—reference group; BMI—body mass index; oGTT—100 g oral glucose tolerance test; SAV—single abnormal value; GDM—gestational diabetes.

**Table 4:** Cox-regression analysis for the development of type 2 diabetes mellitus according to study groups for women without obesity.

	aHR	95% CI	*p* Value
Maternal age, years	1.062	1.035–1.090	<0.001
Maternal hypertension	1.946	0.481–7.881	0.351
Normal glucose tolerance	***		
SAV late oGTT	3.300	1.048–10.398	0.041
SAV on-time oGTT	3.096	1.139–8.415	0.027
GDM late oGTT	7.708	3.140–18.920	<0.001
GDM on-time oGTT	8.939	4.503–17.743	<0.001

Results are presented as Hazard ratios (95% confidence interval), adjusted for maternal age and BMI. BMI—body mass index; oGTT—100 g oral glucose tolerance test; SAV—single abnormal value; GDM—gestational diabetes. *** The normal glucose tolerance is the reference value. The other parameters were compared to it.

**Table 5:** Cox-regression analysis for the development of type 2 diabetes mellitus according to study groups for women with obesity.

	aHR	95% CI	*p* Value
Maternal age, years	1.038	1.021–1.056	<0.001
Maternal hypertension	1.863	1.068–3.250	0.028
Normal glucose tolerance	***		
SAV late oGTT	0.999	0.445–2.244	0.999
SAV on-time oGTT	1.957	1.181–3.242	0.009
GDM late oGTT	2.091	1.035–4.226	0.040
GDM on-time oGTT	2.727	1.668–4.460	<0.001

Results are presented as Hazard ratios (95% confidence interval), adjusted to maternal age and BMI. BMI—body mass index; oGTT—100 g oral glucose tolerance test; SAV—single abnormal value; GDM—gestational diabetes. *** The normal glucose tolerance is the reference value. The other parameters were compared to it.

## Data Availability

Data may by available upon request.
